# Population-level impacts of reducing artificial light at night across an entire migratory corridor

**DOI:** 10.1098/rsbl.2026.0304

**Published:** 2026-09-09

**Authors:** Brendan M. Lehman, Benjamin P. Burford, Thomas R. Nelson, Nicholas J. Demetras, Cyril Michel

**Affiliations:** ^1^ Institute of Marine Sciences, University of California, Santa Cruz, CA, USA; ^2^ National Marine Fisheries Service, National Oceanic and Atmospheric Administration, Santa Cruz, CA, USA; ^3^ Department of Environmental Science and Policy, George Mason University, Fairfax, VA, USA

**Keywords:** ALAN, artificial illumination, fish, predator–prey interactions

## Abstract

Artificial light at night (ALAN) is a widespread anthropogenic pollution that poses challenges for wildlife in nearly every ecosystem on planet Earth. Although there has been substantial progress on understanding the effects of ALAN on animal physiology and behaviour, few studies have estimated its population-level impacts in the wild, leaving the potential conservation benefits of ALAN reduction unresolved. Here, we evaluated the population-level effect of ALAN reduction through its impact on juvenile Chinook salmon (*Oncorhynchus tshawytscha*) vulnerability to predation during oceanward migration through California's Sacramento–San Joaquin Delta (Delta). Removal of all ALAN sources was conservatively predicted to allow up to 1123 additional successful migrants per million migration attempts, depending on the baseline rate of through-Delta migration success and phototaxic behaviour. Reducing the impact of ALAN in this system could have small but worthwhile benefits for salmon at a cost and degree of contention that is trivial compared with many other habitat restoration actions.

## 1. Introduction

Artificial light at night (ALAN) is a physical disruption to the landscape that affects the behaviour of wildlife [1,2]. Organisms interact with ALAN on multiple scales—both through direct glare, as well as general skyglow. At a local scale, artificial lights create a patchwork of illuminated and dark areas that animals may be attracted to or repelled by. Transiting lighted areas can lead to increased mortality owing to increased predator–prey interactions, as well as indirect mortality by disrupting feeding behaviour, or from exhaustion such as from circling lights [3].

The effects of ALAN on Pacific salmon (*Oncorhynchus* spp.) are relatively well-studied compared with other fish species [4,5], probably owing to their cultural and economic importance, coupled with substantial fisheries management on the relatively affluent west coast of North America. Juvenile salmon are exposed to a suite of predators during their seaward migrations in most west coast systems. As a result, they generally move at night and exhibit diel vertical migration presumably to minimize the likelihood of being eaten [6–8]. The addition of ALAN may remove or reduce dark refuge habitat, probably increasing predation mortality of outmigrants.

Many freshwater fish can visually detect prey at extremely low light resembling ambient moonlight, and predatory fish often have a relative advantage over prey at light levels well below 1 lx [9–11]. Several laboratory studies have observed an increase in predation on juvenile salmon under low light conditions resembling artificially lit shorelines at night [12–14]. But owing to the challenges of observing predation in aquatic environments, only a few have documented it in the field [15,16]. Artificially lighted areas may be increasing predation by aggregating predators and/or prey [17]. Nelson *et al.* [18] corroborated these results, observing increased predator aggregation under illuminated versus dark areas coupled with increased predation rates. Pérez Vega *et al.* [19] recently proposed a conceptual model for how artificial light may affect Atlantic salmon (*Salmo salar*) and European silver eel (*Anguilla anguilla*) populations owing to predation and migratory delay. To date, however, no study has determined population-level consequences that may result from elevated predation rates on fish owing to the influence of individual sources of ALAN by combining results from experimental behavioural studies with empirical measurements of ALAN across a landscape.

Our objectives were to estimate population-level benefits of reducing artificial light for migrating juvenile salmon in a large estuarine system. We surveyed all migration corridors in the system for ALAN, created a high-resolution inventory of light sources, and interpolated the two-dimensional (2D) distribution of artificial light levels across the entire estuary. To identify a relationship between ALAN and predation rates above baseline mortality in this system, we used data from Nelson *et al.* [18]. This field experiment was conducted in the same estuarine system as our survey, and quantified predation rates under varying levels of ALAN using live juvenile salmon tethered to floating buoys. Finally, we combined the model output and ALAN survey data to estimate how many more juvenile salmon may survive seaward migration through this system under different behaviours and different baseline survival rates. We discuss a method for objectively identifying areas where salmon may benefit most from reduction of artificial lighting.

## 2. Material and methods

### (a) Study system

The California Delta (hereafter referred to as the Delta) is the largest freshwater estuary on the west coast of North America with over 1100 km of inter-crossing waterways and sloughs, and has been heavily modified since the late 19th century [20]. The Delta hosts a variety of migratory fish species, including Chinook salmon (*Oncorhynchus tshawytscha*), whose populations are dwindling [21]. Habitat loss owing to upstream dams and water exports is thought to be the proximal driver of salmon decline in this watershed [22]. However, one of the bottlenecks preventing salmon recovery in California is high mortality rates of juveniles migrating through, or attempting to rear in, the Delta as a result of predation by introduced fish species such as striped bass (*Morone saxatilis*) and black bass (*Micropterus* spp.) [23–25]. Because eradicating these predatory fish species is not feasible [26], there is a need to identify habitat restoration actions that can make conditions more favourable for native species such as Chinook salmon.

### (b) Data collection

We traversed all major Delta waterways (approx. 400 km) in a small outboard boat with GPS and an International Light Technologies ILT2400 light meter mounted on a custom bowsprit that extended in front of the boat. To standardize measurements, we fixed our sensor vertically, as this is the only option that does not introduce subjective decisions to be made when collecting data. Although light attenuates quickly in water, the Delta is relatively shallow (generally less than 5–10 m deep).

Surveys occurred October to November 2021, over the course of eight nights concurrent with a new moon and a range of clear to cloudy conditions. Data collection began 1.5 h after sunset. In unilluminated areas, we traversed the middle of the river channel at full speed with the sensor continuously recording once per second. These measurements allowed us to quantify background illumination owing to stars and skyglow from nearby cities, as well as to subtract from measurements made at ALAN point sources if necessary.

At each light source that cast direct glare onto the water, we collected information such as structure type (e.g. streetlamp, bridge, etc.), colour, number of bulbs, and whether it was a public or private installation. We then conducted a minimum of three transects through the illuminated area with the light meter recording at 1-s intervals. One transect was made by passing as close to the light(s) as possible, and one either at the edge of the area of influence, or in cases where light extended across the channel, on the opposite bank. For areas with a complex mosaic of light sources, we made additional passes haphazardly until there was enough spatial coverage to interpolate a fair representation of the area.

### (c) Inventory and interpolation

**Figure 1.**
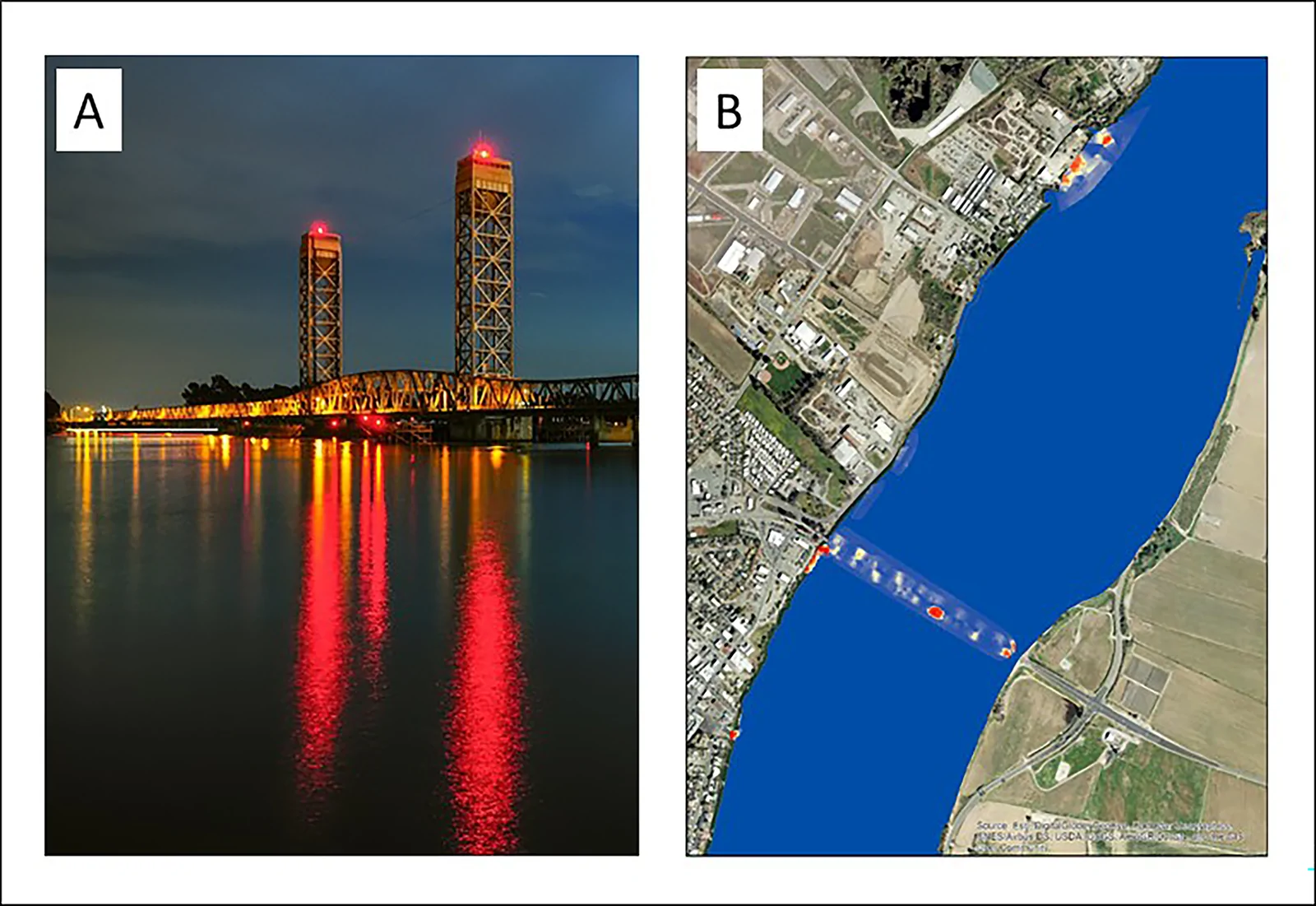
(A) ALAN cast by lighting on a busy two-lane highway bridge spanning a section of the 50-km migratory corridor (photo credit: Darvin Atkeson). (B) Surface illumination intensity of the aforementioned bridge, as well as streetlamps, several commercial docks and a public fishing pier in the vicinity of a small settlement and industrialized shoreline.

We generated a 2D map of surface light using ArcMap 10.6.1 (ESRI) (figure 1). We created a polygon shapefile representing relevant waterways to serve as a boundary and used the Inverse Distance Weighted tool to interpolate light levels with a 3-m cell size, resulting in a raster layer of light intensity across the region at 3 m^2^ resolution.

### (d) Salmon survival estimates

To estimate the impact of light intensity on salmon survival, we used data and the best supported model from a previous field experiment that occurred within our survey area in the same estuarine study system. Nelson *et al.* [18] observed changes in predator density and quantified predation rates across a gradient of illumination intensities, which encompassed intensities observed in our survey, by installing light fixtures along riverbanks at five sites that were broadly representative of nearshore habitats in the Delta. The authors used underwater sonar imaging cameras to quantify predator densities and deployed predation event recorders (PER; [27]) baited with juvenile Chinook salmon smolts to measure changes in predation rates at different light levels. PERs are small, GPS-enabled floating buoys outfitted to collect relevant environmental covariates. They are tethered with a live bait fish tied to a length of fishing line and repeatedly drifted through the study area over time. When a predator attempts to eat the bait, a magnet attached to the line is pulled and the PER records the time and location of the event.

We accessed this dataset from the Environmental Data Initiative repository (‘per_ALAN.csv’; [28]). We used a Cox proportional hazard model [29] to estimate relative predation risk as a function of ALAN and other environmental covariates collected during the study. Cox proportional hazard models are time-to-event models commonly used in clinical trials in the medical literature; in this situation, the event is predation as measured by PERs. On the basis of the authors’ finding that ALAN only affected predation rates several hours after sunset, we subset the data to samples collected approximately 3–5 h past sunset, and only for PER deployments that drifted through artificially illuminated areas. We modelled instantaneous predation risk using the best supported model from Nelson *et al.* [18], which was a function of surface lux intensity, predator density and time past sunset. Linear predictions for the effect of light were made at mean predator density and time past sunset. We ran the Cox model and checked model assumptions, using the Surv, coxph, and cox.zph functions from the survival (v. 3.3-1; [30]) package, within the programme R (v. 4.2.1; [31]).

Survival rates of juvenile salmon migrating through the Delta are commonly estimated using acoustic telemetry [32–36] and used as a monitoring metric for population management. To explore the potential benefits of ALAN mitigation on through-Delta survival rates, we performed extrapolation scenarios focused on a 50-km reach of the Sacramento River mainstem channel—the primary migratory pathway for Chinook salmon in the Delta—across hypothetical baseline survival rates between 0% and 100%. The experimental prey in Nelson *et al.* [18] were unable to swim freely, so the phototaxic behaviour of juvenile salmon in this system was not determined. We provide estimates of this behavioural uncertainty by assuming that prey are either attracted to, repelled by, or indifferent to artificial light (positive, negative, or neutral phototaxis, respectively).

Using ArcGIS, we created a river channel centreline feature and then perpendicular transect lines every 10 m, spanning wetted areas, and then split into equally spaced points 1 m apart. Each point within a line is assigned the baseline hazard rate per 10 m (HR_baseline_), which is a function of overall survival through the region (ϕ_baseline_) and the number of reaches (*n*reaches):

$$HR_{baseline}=-log(\phi {baseline}_{}^{1/nreaches})$$

Next, we assign a new survival rate for each point (φ_illuminated_) accounting for ALAN, which is a function of the baseline hazard rate (HR_baseline_) and the predation model output at the relevant lux level for each point (HR_illuminated_):

$$\begin{array}{r} \varphi_{illuminated}=e^{-1*\left(HR_{baseline}*HR_{illuminated}\right)} \end{array}$$

Each river reach then receives an adjusted survival rate (ϕ_adjusted_) based on the additional risk of ALAN. For neutral phototaxis, this is the mean of all points along the respective transect line. For positive or negative phototaxis, it is the minimum or maximum ϕ_illuminated_ within each reach, respectively. Finally, the product of every reach's ϕ_adjusted_ produces a cumulative survival rate for the entire migratory corridor accounting for the consequences of artificial lighting on predation rates.

Five state and federal fish hatcheries in the Central Valley produce approximately 30 million fall-run Chinook salmon smolts annually [37] and several more million late-fall, winter, spring-run Chinook, and steelhead trout (*Oncorhynchus mykiss*), in addition to the more variable wild-spawning production of all runs. Therefore, an intuitive way to conceptualize the relative effect of removing sources of ALAN on the population is to estimate the total number of additional surviving fish for every 1 000 000 salmon smolts that transit the region on their seaward migration:

$$=1000000*\phi {}_{baseline}-1000000*\phi_{adjusted}$$

Using this framework, the effect depends on the input baseline survival, so we calculated this for ϕ_baseline_ between 0 and 1. We then divided the additional survivors by the number of baseline survivors to estimate the percentage increase of fish that survive their migration at any given baseline survival rate.

## 3. Results

Illumination from skyglow in most of the Delta was minimal, and we found no regions where background illumination levels were higher than starlight (generally <0.006 lx). We did identify 151 locations with sources of illumination that cast light directly onto waterways. These were present in all regions, including both mainstem and distributary channels. The affected areas ranged in size from 47 m^2^ to 115 226 m^2^ (*M* = 7458, s.d. = 14 939) and had maximum measured brightness at the water surface ranging between 0.012 lx and 116.8 lx (*M* = 8.4, s.d. = 19.9). The proportion of channel width that light was detectable for each site ranged from 0.006 to 1 (*M* = 0.0362, s.d. = 0.278).

For the subset of data collected 3–5 h after sunset, and in illuminated reaches, relative predation risk was positively correlated with illumination intensity (*p* = 0.003) and predator density (*p* = 1.5 × 10^–13^) (table 1). Time past sunset was not significantly correlated with predation risk (*p* = 0.057) under these conditions. The model predictions for illumination intensity, based on the mean predator density within the illumination treatment study reaches, yielded a positive intercept (lp = 0.0262) and an increase of 2.95 × 10^–4^ for each unit 0.01 lx increase in brightness. Our model satisfied all proportional hazard assumptions.

**Table 1.** Results from a Cox proportional hazard model.

data	coefficient	hazard ratio	scaled hazard ratio	s.e.	*Z*	*p*
lux	0.029	1.030	0.009	0.010	3.017	0.003
pred dens	44.156	1.502E+19	5.979	5.979	7.385	0.000
minutes	–0.006	0.994	0.252	0.003	–1.902	0.057

NOTE: Predictor variables are illumination intensity (lux), predator density (no. of fish >200 mm m^–3^), and minutes past sunset (min). The hazard ratio is the change in the relative predation risk per unit increase of each predictor variable.

**Figure 2.**
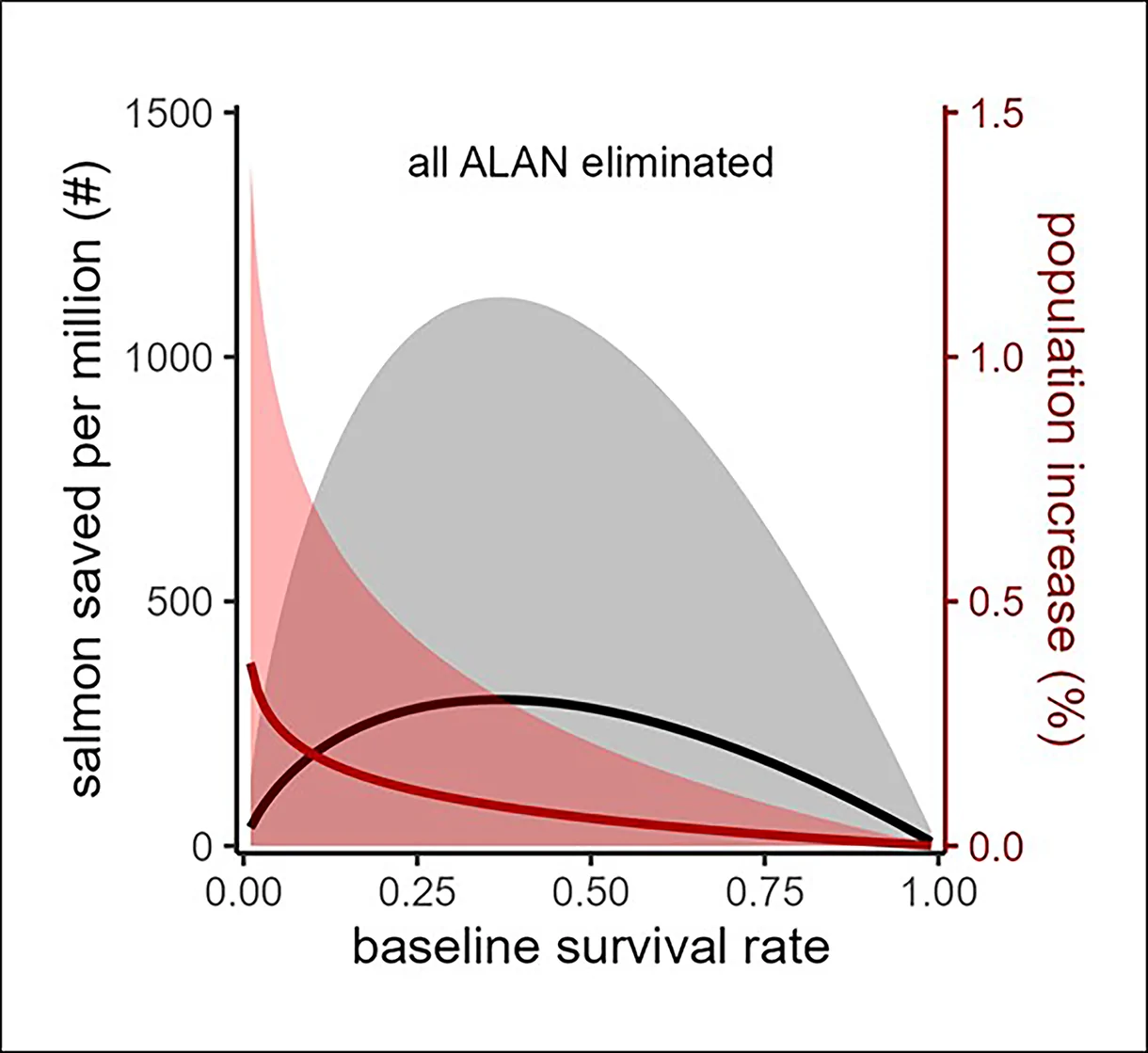
Black line shows the number of additional juvenile Chinook salmon (per million migrants) predicted to survive migration through the 50-km migratory corridor if all ALAN was eliminated across potential baseline survival rates under the neutral phototaxis scenario, where salmon are exposed to the mean light intensity of each cross-section of the river channel, whereas red shows these numbers expressed as a percentage of population increase. The upper extent of the ribbons shows the largest predicted impact of ALAN elimination under the positive phototaxis scenario where salmon are attracted to light, whereas the lower extent shows the smallest predicted impact under the negative phototaxis scenario, which is nearly zero owing to salmon avoiding lighted areas.

For Chinook salmon, the probability of successful through-Delta migration averages between 0.32 and 0.70, depending on the conditions encountered [36]. Under these rates, we found ALAN elimination could result in 763–1123 additional successful migrants per million migration attempts (i.e. 0.08–0.11% population increase, respectively), assuming that juvenile Chinook salmon exhibit positive phototaxis (figure 2), which available literature suggests is the case [38]. In terms of a relative population increase, ALAN elimination had the greatest impact when baseline survival was below average (<0.32), which can occur during drought years in California.

## 4. Discussion

Our results suggest that eliminating all ALAN in the Delta could result in a 0.11% increase in surviving migrants for populations of imperilled Chinook salmon. Although this increase may seem trivial at face value, the seaward migration of Central Valley salmon is typically just days to weeks of their 3- to 4-year lifespan. This short period has been identified as the primary bottleneck to population recovery, with mortality rates per unit time orders of magnitude higher during seaward migration than the rest of their lifespan [39]. Under drought conditions, through-Delta survival can be <2%, making replacement rates for the next generation unachievable [40].

**Figure 3.**
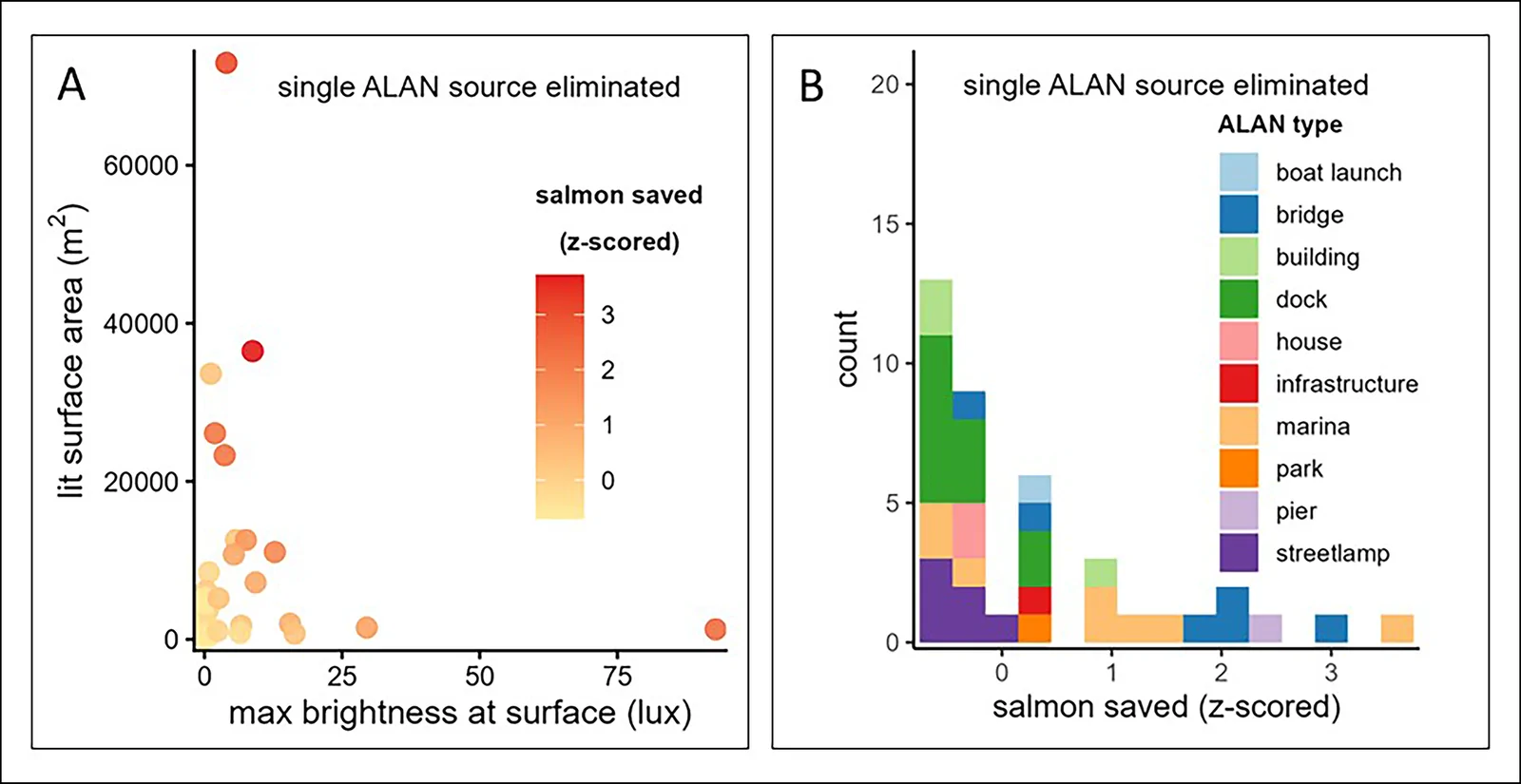
The relative number of additional salmon saved per million (z-scored) predicted to survive through-Delta migration under a 50% survival scenario if single sources of ALAN were eliminated, shown as a function of (A) ALAN source brightness and illuminated area or (B) ALAN source type.

Our survey revealed that the Delta is a fairly dark place at present, which is one reason that population-level impacts of ALAN elimination were projected to be relatively low. Although not absent of skyglow, background illumination levels during this survey, even on cloudy nights, were lower than the resolution of our lux meter, and probably negligible below the water's surface. However, the locations lit by direct glare from local light sources are most probably affecting fish behaviour to some degree. The brightness, size and proportion of channel spanned by direct glare varied by orders of magnitude, but nearly all cases increased localized brightness at the surface by 10-fold compared with background starlight. In particular, we found that a few locations may disproportionately increase predation on juvenile salmon in this system (figure 3A), and a cost–benefit analysis could identify relevant targeted restoration actions. The sources of light were variable, and we found no trends in the use type or ownership (i.e. private, public) for the most impactful (figure 3B).

Another reason our model projected a relatively low impact of ALAN reduction on salmon populations is that our estimates are based on simplified assumptions about fish distribution and movement. Although ALAN is only relevant during nighttime (less than half the diurnal cycle for most of the year at this latitude), studies have found most juvenile salmon migrate at night in the Delta [6,41]. Therefore, for this exercise, we assumed that all fish experienced the effects of the illuminated areas they transited via positive, negative or neutral phototaxis. Anderson *et al.* [42] proposed a mean free-path length model for predation on migrating salmon where the likelihood of being eaten is a function of both distance travelled, as well as time spent in the habitat. Juvenile salmonids, as well as larger predatory fish, are known to aggregate under artificial lights during the nighttime [3,16–18,38,43], which increases the time spent in lighted areas and delays migration, thus increasing the exposure level of prey to predators in both space and time. Previous *in situ* field studies have used experimentally illuminated areas, but permanent light installations may result in predators cueing in and specializing in capturing disoriented prey, creating predation hotspots [44]. Sridharan *et al.* [45] found that juvenile salmon exhibit both upstream and downstream movements when migrating through the Delta, potentially exposing them to illuminated areas more than once. These caveats suggest our estimates of the population-level impact may be conservative. Furthermore, there may be light thresholds above which there is a paradigm shift of how organisms interact with the environment and with each other, which is a worthwhile avenue of future research for estuarine fishes in anthropogenically altered environments.

Although the effects of ALAN on predation of juvenile salmon are secondary to other factors like temperature [24,25], water diversions and exports [46,47] and invasive species [48,49], reducing ALAN is a relatively low-cost habitat restoration action that only needs to be paid once, relatively less likely to cause political backlash or conflict with human needs, and may even result in energy cost savings in the long run. Furthermore, because the relative benefits are greater when baseline survival is low, reducing ALAN may provide a small but meaningful buffer during drought years when survival is poor. Additionally, ALAN presumably disrupts countless other animals’ behaviour and interactions, so restoration actions could benefit other species or ecosystem processes.

To our knowledge, this was the first survey of artificial light that covered the entire landscape of a wild animal population, or at least its obligate migratory corridor, at a resolution fine enough to quantify the illumination level that an individual would experience at a precise location. This case study is unique to fish migration through a riverine corridor. However, there are opportunities to apply our results to other species and ecosystems. A similar strategy for a 2D system, such as patchy light sources dispersed across a terrestrial environment, could be to conduct illumination surveys within randomized grids and extrapolate the distribution across a landscape [50].

Although skyglow has received increasing attention in recent years, scant data on the distribution of individual artificial light sources have been collected. However, light meters are inexpensive, and in non-urbanized areas where light is coming from discrete sources, surveys can be relatively simple to conduct. This type of information will be increasingly useful for allocating restoration dollars or designing new regulations aimed at reducing the impact of ALAN on natural systems.

## Data Availability

Data and original code have been deposited at the Environmental Data Initiative Data Portal and are publicly available as of the date of publication at https://cvpia.scienceintegrationteam.com/cvpia-sit/resources/data-assets. Data can also be found here: https://doi.org/10.6073/pasta/9fb1e8cd0d51a39018fd846dd54ef9b3. Any additional information required to re-analyse the data reported in this paper is available from the lead contact upon request.
